# Malaria in Pregnancy: From Placental Infection to Its Abnormal Development and Damage

**DOI:** 10.3389/fmicb.2021.777343

**Published:** 2021-11-11

**Authors:** Caroline Lin Lin Chua, Sebastian Kah Ming Khoo, Jun Long Ernest Ong, Gaurav Kumar Ramireddi, Tsin Wen Yeo, Andrew Teo

**Affiliations:** ^1^School of Biosciences, Taylor’s University, Subang Jaya, Malaysia; ^2^Lee Kong Chian School of Medicine, Nanyang Technological University, Singapore, Singapore; ^3^Department of Medicine, Monash University, Victoria, VIC, Australia; ^4^National Center for Infectious Diseases, Singapore, Singapore; ^5^Department of Infectious Diseases, Tan Tock Seng Hospital, Singapore, Singapore; ^6^Department of Medicine at Royal Melbourne Hospital, Peter Doherty Institute, University of Melbourne, Melbourne, VIC, Australia

**Keywords:** low birth weight, preterm birth, malaria, pregnancy, *Plasmodium falciparum*, syncytiotrophoblast, placental insufficiency, fetal growth restriction

## Abstract

Malaria remains a global health burden with *Plasmodium falciparum* accounting for the highest mortality and morbidity. Malaria in pregnancy can lead to the development of placental malaria, where *P. falciparum*-infected erythrocytes adhere to placental receptors, triggering placental inflammation and subsequent damage, causing harm to both mother and her infant. Histopathological studies of *P. falciparum*-infected placentas revealed various placental abnormalities such as excessive perivillous fibrinoid deposits, breakdown of syncytiotrophoblast integrity, trophoblast basal lamina thickening, increased syncytial knotting, and accumulation of mononuclear immune cells within intervillous spaces. These events in turn, are likely to impair placental development and function, ultimately causing placental insufficiency, intrauterine growth restriction, preterm delivery and low birth weight. Hence, a better understanding of the mechanisms behind placental alterations and damage during placental malaria is needed for the design of effective interventions. In this review, using evidence from human studies and murine models, an integrated view on the potential mechanisms underlying placental pathologies in malaria in pregnancy is provided. The molecular, immunological and metabolic changes in infected placentas that reflect their responses to the parasitic infection and injury are discussed. Finally, potential models that can be used by researchers to improve our understanding on the pathogenesis of malaria in pregnancy and placental pathologies are presented.

## Introduction

Malaria is a blood-borne disease caused by *Plasmodium* spp., with *Plasmodium falciparum* (*P. falciparum*) being the most deadly species (World Health Organization [WHO], 2020). Pregnant women, especially first-time mothers, are at high risk of severe malaria due to *P. falciparum*, hence *P. falciparum-*related malaria in pregnancy (MiP) will be the focus of this review. Long-term childhood exposure to the parasites can result in the development of protective antibodies; however, first-time pregnant mothers become susceptible again (Teo et al., 2016). Their susceptibility can be attributed to the *P. falciparum* erythrocyte membrane protein-1 (PfEMP1), a major variant surface antigen displayed on the surface of *P. falciparum*-infected erythrocytes (IEs) that serves as an adhesin (Lennartz et al., 2019; Wang et al., 2021). Each parasite has up to 60 *var* genes that encode the PfEMP1 molecules, and only a single variant is expressed at any one time (Smith et al., 2001). Switching of this *var* gene expression enables *P. falciparum* to evade host immunity. Consequently, IEs can sequester in organs and avoid splenic clearance, which then promotes inflammation and/or microvasculature obstruction (Biggs et al., 1991; Dondorp et al., 2008; Chua et al., 2021b). VAR2CSA is a PfEMP1 molecule that is expressed during MiP, and it mediates the binding of IEs to placental receptors such as chondroitin sulfate A found on placental syncytiotrophoblast (SCT) (Salanti et al., 2003; Tuikue Ndam et al., 2005). As a result, IEs accumulate within the placenta, triggering an inflammation in the placental intervillous spaces; the infected and inflamed placenta is commonly termed as placental malaria (PM). PM is particularly common amongst first-time pregnant women, due to their lack of immunity against placental-binding IEs, and this is likely to adversely impact the placenta, leading to poor outcomes in MiP (Fried et al., 1998; Staalsoe et al., 2001; Tran et al., 2020). However, our understanding on the physical and physiological changes to the infected placentas during PM is still rather limited, mainly due to the invasive nature of studying placental tissues during pregnancy and the limitations associated with existing animal models of MiP.

In this review, we will first provide a general overview of a successful pregnancy, followed by a detailed discussion on MiP and its deleterious impact on the placenta. The different immunological, molecular and metabolic changes in the infected placentas following parasitic infection, which are subsequently linked to placental injury and its dysregulated physiology, will be reviewed. We will include findings from several established rodent models of MiP which have enhanced our understanding on the topic, albeit there are differences between humans and rodents, as described in Table 1. Lastly, we discuss potential models that can be used by researchers to better understand the pathogenesis of MiP and the mechanisms underlying placental damage and injury in this disease.

**TABLE 1:** Comparison between humans and rodent models of malaria in pregnancy.

	**Human**	**Rodent**	**References**
Parasitemia	• Infection is through the bite of an infective mosquito, transmitting sporozoites during a blood meal. This triggers a complex pathway of inflammation; however, case of extreme parasitemia is rarely observed in pregnant women. This is presumably due to the use of effective antimalarials and the acquisition of protective antibodies during subsequent pregnancies.	• Infection is often through blood-stage inoculation; this may trigger different pathways of inflammation. Blood-stage infection results in high parasitemia that is often lethal. Because of the shorter duration of pregnancy, immunity during pregnancy is harder to investigate.	Humans protective antibodies—(Fried et al., 1998; Ndam et al., 2015). Mouse models—(Poovassery and Moore, 2006; Neres et al., 2008) Mouse models of immunity—(Marinho et al., 2009; Megnekou et al., 2009)
Placental anatomy	• Placenta is comprised of tree branch-like villi. • Placental invasion is mediated by extravillous trophoblast cells that are highly invasive. • Placenta is hemomonochorial, where maternal blood is in direct contact with fetal villi, separated only by the syncytiotrophoblast layer.	• Placenta is comprised of labyrinth villous structure. • Placental invasion is mediated by trophoblast giant cells that are modestly invasive. • Placenta is hemotrichorial, where maternal blood is separated from fetal blood via three thin layers of trophoblastic cells (cytotrophoblasts and two layers of syncytiotrophoblasts).	Differences in placental anatomy—(Takata et al., 1997; Georgiades et al., 2002; Cline et al., 2014; Schmidt et al., 2015; Soncin et al., 2015)
Parasite antigens	Parasites that infect pregnant women express *var* genes coding for VAR2CSA proteins, which are expressed on IEs and are responsible for binding to placental receptors such as chondroitin sulfate A.	Rodent parasites express antigens transcribed by the multigenic *Plasmodium* interspersed repeats (*pir*) superfamily. Their function in MiP and PM is unknown.	VAR2CSA—(Salanti et al., 2003; Tuikue Ndam et al., 2005; Duffy et al., 2006) *pir* multigene—(Janssen et al., 2004; Hall et al., 2005)
IE sequestration	• IEs can sequester in placental intervillous spaces, mediated by their binding to the syncytiotrophoblast, and this is the hallmark of placental malaria. • Infection may be “hidden” in the placenta, as higher parasite density has been found in the placenta compared to maternal peripheral circulation.	• Evidence of IE binding to the placenta is less prominent, likely due to the lack of VAR2CSA homologs. However, IEs are often observed to accumulate in the placenta. • High parasite density observed in the placenta may be due to overall high parasitemia in maternal peripheral blood, resulting in the circulation of parasites into the placenta.	Human studies on placental sequestration—(Fried and Duffy, 1996; Ordi et al., 1998) Mouse models of placental parasite accumulation—(Neres et al., 2008; Sharma et al., 2016; Morffy Smith et al., 2019)
Placental inflammation and pathology	• Excessive immune cell accumulation in placental intervillous spaces, termed intervillositis, has been observed in chronic cases, and is associated with poor placenta development and birth outcomes. • Human pregnancy has a longer gestation period. Thus, inflammation at different trimesters which disrupt the cytokines/chemokines balance may disrupt placental development or/and function, depending on the stage of pregnancy.	• Mice challenged with *P. chabaudi* sporozoites displayed little evidence of immune cell accumulation; however, when challenged with blood-stage *P. chabaudi* or *P. berghei*, massive accumulation of immune cells has been observed. • Experimental infection of mice often takes place later in pregnancy when the placenta is fully developed, as infection early in pregnancy results in mortality and loss of pregnancy.	Intervillositis in human—(Ordi et al., 1998; Rogerson et al., 2003b; Muehlenbachs et al., 2010) Mononuclear cells infiltration in mice—(Neres et al., 2008; Poovassery et al., 2009; Sarr et al., 2015; Rahmah et al., 2019) Lack of monocytes and macrophages accumulation in placentas of mice—(Poovassery and Moore, 2006; Sharma et al., 2016)

## A Successful Pregnancy

A successful pregnancy requires proper development of the placenta and its sustenance throughout pregnancy. In the first trimester, T_*h*_1 pro-inflammatory responses promote proper tissue remodeling and angiogenesis; dysregulated immune responses have been associated with increased risk of early pregnancy failures (Wang et al., 2020). Subsequent shift toward a T_*h*_2 environment during the second trimester, characterized by increased anti-inflammatory cytokine levels and expansion of regulatory T cells, allows for rapid fetal growth and prevents fetal rejection (Aluvihare et al., 2004; Wang et al., 2020). At the end of pregnancy, there is a shift back to the pro-inflammatory environment with increased IL-1β, IL-6, and IL-8 expression, which are essential for parturition (Christiaens et al., 2008; Rinaldi et al., 2017). However, elevated T_*h*_1-type responses in the third trimester can increase the risk of pre-term birth (Romero et al., 2014; Fried et al., 2017).

Various immune tolerance mechanisms in the placenta play crucial roles to prevent fetal rejection. For example, placental trophoblasts do not express the classical major histocompatibility complex (MHC) molecules, which can otherwise trigger a pro-inflammatory immune cascade. Instead, they widely express the non-classical human leukocyte antigen (HLA)-G and HLA-E molecules that protect the placenta from CD8^+^ T cell and decidual natural killer cell cytotoxicity (Tersigni et al., 2020; Xu et al., 2020). The placenta can also secrete exosomes with immuno-regulatory functions, such as inducing the differentiation of macrophages to display characteristics of decidual cells that play important roles at the maternal-fetal interface (Bai et al., 2021). In addition, Hofbauer cells, which are fetal-derived placental macrophages in the chorionic villi, display M2 phenotype and express IL-10 (Yang et al., 2017). IL-10 is an anti-inflammatory cytokine expressed throughout pregnancy, with higher expression levels detected during first and second trimesters, and the lack of this cytokine was associated with poor pregnancy outcomes such as growth restriction and preterm birth (Cheng and Sharma, 2015). However, clinical studies and rodent models, increased IL-10 levels at delivery have been associated with poor birth outcomes, suggesting possible impairment in parasite clearance that may contribute to poor outcomes (Megnekou et al., 2013, 2015a). Overall, the dysregulation of either T_*h*_1 or T_*h*_2 responses during pregnancy may contribute to adverse outcomes and maintaining a balance between pro- and anti-inflammatory immune responses is crucial for a healthy pregnancy.

## The Development of the Human Placenta

**FIGURE 1:**
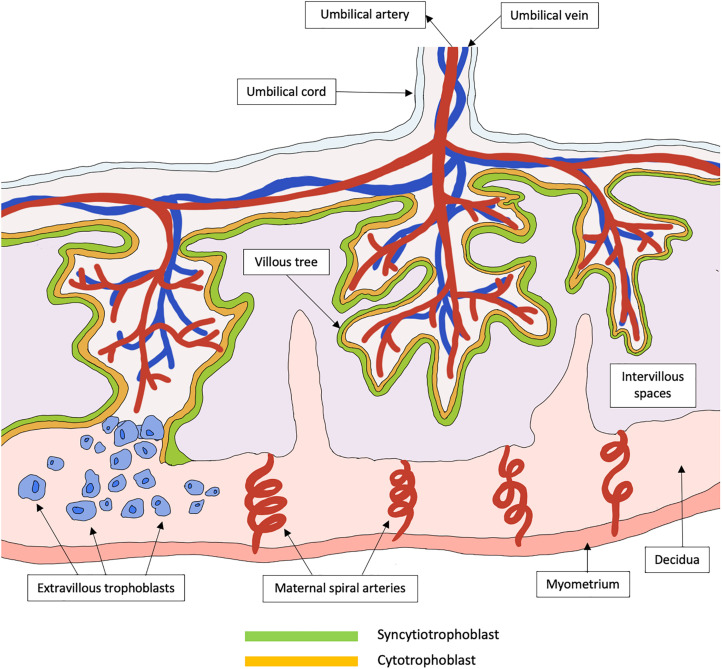
Schematic representation of a healthy functional unit of the human placenta. The placenta is a specialized organ that is primarily involved in the exchange of metabolites between the mother and her fetus. In the early trimester, the trophoblast differentiates to form primary syncytium that further divides into the cytotrophoblast and syncytiotrophoblast. The syncytiotrophoblast will then expand to form a continuous layer of tree-like villous that are in contact with maternal blood in the intervillous spaces. These villous trees greatly increase the surface areas available for the exchange of metabolites. Cytotrophoblasts can further differentiate into extravillous trophoblasts that invade the decidua, which is the maternal uterine tissue, to promote immune tolerance between the mother and her fetus. The extravillous trophoblasts also migrate up the maternal spiral arteries and promote spiral artery remodeling, to form large vessels of low resistance that is required to sustain a healthy pregnancy.

The placenta is a highly specialized organ that regulates the exchange of metabolites between fetal blood in the chorionic villi and maternal blood within the intervillous spaces. Defective placental development can lead to placental insufficiency, contributing to fetal growth restriction (FGR), miscarriage and stillbirth (Brosens et al., 2011). Critically, placental development is a complex and yet delicate process. In the first trimester, the trophoblast, a specialized group of cells, differentiates to form blastocyst, and the outer layer termed trophectoderm fuses to the uterine endometrium to form the primary syncytium. The primary syncytium then further divides and differentiates into the cytotrophoblasts (inner core) and SCT (outer layer). Around 6–8 weeks after implantation, the SCT rapidly expands, forming branching villous trees that invade maternal intervillous spaces and endometrial glands, subsequently allowing the perfusion of intervillous spaces with maternal blood. Toward the second trimester, the cytotrophoblasts continue to expand, giving rise to extravillous trophoblasts that will further invade the decidua and maternal uterine spiral arteries (Figure 1). The uterine spiral arteries then undergo extracellular remodeling through degradation and rebuilding, a process which is critical for ensuring adequate blood supply to the placenta during pregnancy. This complex process involves various immunological cells such as decidual macrophages, regulatory T cells and uterine natural killer cells (Staff et al., 2020), as well as angiogenic, vascular, and placental growth factors (Hanna et al., 2006; Lash et al., 2006).

## Placental Pathologies During Malaria in Pregnancy

### Dysregulated Placental Vascularization and Angiogenesis

Bilateral uterine artery notching and concomitant vascular resistance are signs of disruption to the utero-placental hemodynamics and these have been observed in *P. falciparum*-infected placentas (Dorman et al., 2002). Placental vascularization begins as early as eight gestational weeks (GW) (Leijnse et al., 2018), hence infection during and after this period may affect the development of placental vasculature and its blood flow. In placentas from Tanzanian women, MiP before 15 GW was associated with decreased volume of transport villi and increased diffusion distance in diffusion vessels, suggesting impairment of placental vascular development (Moeller et al., 2019). Furthermore, Doppler ultrasound studies showed that infection before 20 GW in primigravids was associated with increased umbilical artery resistance, an indirect measurement of resistance flow within the placenta (Griffin et al., 2012; Ome-Kaius et al., 2017). A study on a cohort of Malawian pregnant women revealed that MiP infection between 13 and 23 GW was associated with dysregulation of various angiogenic factors and metabolic hormones, which play vital roles in placental vascularization (Elphinstone et al., 2019). Impaired trophoblast differentiation, represented by shallow trophoblast invasion and narrow spiral arteries formation, may contribute to increased umbilical artery resistance and poor placental perfusion, leading to preeclampsia (Roland et al., 2016; Obiri et al., 2020). MiP before 18 GW was associated with reduced trophoblast invasion and migration *in vitro*, suggesting possible reduction in placental blood circulation that might contribute to later pregnancy complications (Abrahams et al., 2005; Umbers et al., 2013). Interestingly, MiP infection in later pregnancy, 32–35 GW, was also associated with increased uterine artery resistance (Dorman et al., 2002). Given that the invasion of trophoblasts is usually complete around 19–20 GW, this pathology is more likely to be due to damaged trophoblasts and/or their impaired function in producing angiogenic factors to sustain exponential fetal growth in the third trimester (Pollheimer et al., 2018). Additionally, in one study, women with PM were reported to be four times more likely to have low placental weight, which is a risk factor for FGR (Singoei et al., 2021). This may be related to the dysregulation of angiopoietins, where women with higher levels of Angiopoietin-2 (Ang-2) were at higher risk of having low placental weight, and in PM, increased levels of Ang-2 in both peripheral and placental blood at delivery have been reported (Silver et al., 2010; McDonald et al., 2016; Singh et al., 2020).

### Irreversible Damage and Abnormal Placental Structure

**FIGURE 2:**
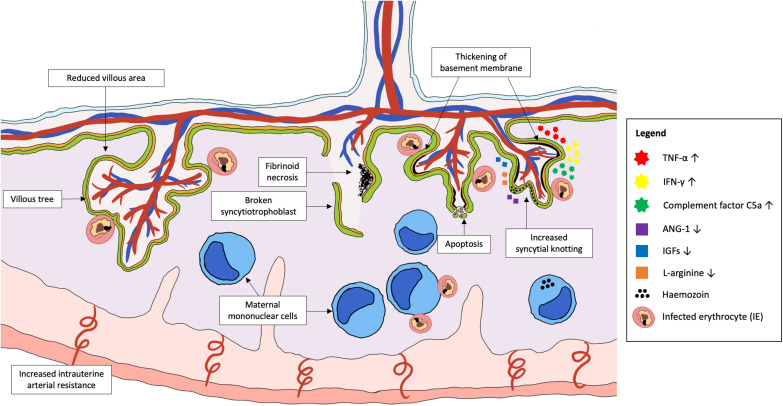
*Plasmodium* parasite infection in the placenta that cause abnormal placental development and damage. Placental malaria is an infection in the placenta by *Plasmodium* spp., commonly *P. falciparum*. Emerging evidence suggests that pregnant women are at the highest risk of PM in the first trimester, which coincides with the period of placental growth and development. Abnormalities in the placenta during PM include permanent damage to placental structures such as broken syncytiotrophoblast, thickening of basement membrane, increased syncytial knotting, increased areas of fibrinoid necrosis and dysregulated apoptosis of trophoblasts. This can result in placental insufficiency that contributes to the increased risk of fetal growth restriction, low birth weight and pre-eclampsia. The pathology is mainly associated with the activation of a local inflammatory response, characterized by the accumulation of activated maternal mononuclear cells within the intervillous spaces. The subsequent pathways leading to placental pathologies include the increased production of pro-inflammatory cytokines, chemokines, and complement proteins, as well as reduced levels of important angiogenic and placental growth factors. Together, these factors may disrupt the development of placenta if infection takes place early in pregnancy, leading to shallow spiral artery development and increased intrauterine arterial resistance.

Irreversible placental damage and abnormal placental structure have been reported in MiP, regardless of the timing of infection. Placentas from women who were infected in their first trimester showed signs of damage at delivery, including reduced transport villi, increased syncytial knotting and increased placental lesions (Crocker et al., 2004; Elphinstone et al., 2019; Moeller et al., 2019). Active infection at delivery was associated with reduced villous area and vascularity, increased basal membrane thickening, syncytial damage, increased syncytial knotting, and fibrinoid necrosis (Crocker et al., 2004; Chaikitgosiyakul et al., 2014). Collectively, the accumulation of fibrin in the intervillous spaces coupled with dysregulation of angiogenesis in placenta can result in inadequate perfusion to the placenta, causing necrosis (Burton and Jauniaux, 2018). Necrotic cell death has been observed in infected placentas, with extensive syncytial breaks in some cases of active PM leading to denudation of the villi, vacuolation, and complete destruction of villous integrity (Crocker et al., 2004). Chronic placental infection at delivery is characterized by increased accumulation of phagocytes in the intervillous spaces and sparse villi numbers, which are likely to reduce nutrient and protein transport across the placenta (Figure 2; Lybbert et al., 2016; Chua et al., 2021a).

Hemozoin, the insoluble product of *Plasmodium* parasites, is commonly observed in the intervillous spaces, either trapped in fibrin or within maternal macrophages (McGready et al., 2002). The presence of hemozoin in infected placentas can further cause placental damage through its immunomodulatory properties. For instance, hemozoin may fuel placental inflammation by reducing parasite clearance, as phagocytes that have ingested hemozoin were shown to have reduced phagocytic capacity and increased cytokine secretion (Tyberghein et al., 2014). Furthermore, an *in vitro* study reported that hemozoin can activate SCT to produce cytokines and chemokines such as Tumor necrosis factor (TNF)-α, IL-8, CCL3, and CCL4; consequently, this may sustain an inflammatory response that contributes to increased inflammatory cell infiltration and fibrinoid deposits in the placenta (Lucchi et al., 2008).

### Apoptosis in the Placenta

Apoptosis is a physiological response that is involved in normal placental growth and development. The apoptosis of placental villi is observed throughout pregnancy, with increasing percentage of apoptosis being reported as the pregnancy progresses to third trimester (Smith et al., 1997b). High rates of apoptosis in the placenta have been associated with several pathological conditions including spontaneous abortion, intrauterine growth restriction, and preeclampsia (Smith et al., 1997a; He et al., 2013). Interestingly, active parasitemia at delivery was not associated with increased placental apoptosis despite being associated with significantly lower birth weights compared to healthy placentas (Crocker et al., 2004). Instead, the study reported reduced apoptosis in placentas with past infections. In contrast, another study showed that apoptotic gene expressions were increased in women with past infections and increased oxidative insults may trigger apoptosis in the placenta (Kawahara et al., 2019). The discrepancies could be because the latter study was based on a larger sample size and the authors performed molecular studies instead of histological analysis to detect apoptosis. In a mouse model of PM, increased oxidative stress, determined by increased levels of malondialdehyde (MDA) and reduced levels of antioxidant enzyme catalase, were associated with enhanced apoptosis in the placentas of mice with active infection (Sharma et al., 2012). Similarly, apoptosis markers were observed in maternal blood and junctional zone trophoblasts in *P. chabaudi-*infected pregnant mice (Sarr et al., 2015). Of note, the junctional zone trophoblasts are cells that are only found in mice placentas; it is unknown if specific types of human trophoblasts similarly undergo apoptosis during infection. In women with PM, similar findings of increased MDA and reduced antioxidant enzyme levels were also reported in placental tissues, suggesting that oxidative stress may contribute to pathophysiology of the placenta; however, whether or not this was mediated through placental apoptosis is unclear (Megnekou et al., 2015b). Further investigations are required to understand the mechanisms of apoptosis and its impact on placental function and outcomes at delivery in MiP.

## Mechanisms Underlying Placental Pathologies in Malaria in Pregnancy

### Disruption to Placental Blood Vessels Formation

Decreased villous area and vascularity were observed in active *P. falciparum* infected-placentas compared to both healthy and previously treated malaria cases (Chaikitgosiyakul et al., 2014). While the underlying mechanism has yet to be fully understood, it is hypothesized that activated maternal complement system during infection may play a role. C5a levels assayed from placental blood correlated negatively to Ang-1 levels, and positively to Ang-2, vascular endothelial growth factor (VEGF) (required for early vessel formation) and sFLt-1 (inhibits angiogenesis by binding to VEGF), suggesting that alterations in angiogenic factor expression may contribute to poor birth outcomes (Conroy et al., 2013; Singh et al., 2020). Increase in the Ang-2:Ang-1 ratio in women with a positive episode of parasitemia during pregnancy has been reported and sustained Ang-2 levels during pregnancy may result in the formation of new blood vessels instead of vessel maturation; this may explain the excessive fetal vessels observed in infected placentas (Leke et al., 2004; Silver et al., 2010). Importantly, in healthy pregnancies, a gradual increase and decrease in Ang-1 and Ang-2 levels, respectively, across pregnancy, enables the initial branching and subsequent maturation of blood vessels to promote normal placental vascular development (Geva et al., 2002). Altered L-arginine level was also proposed as a contributor to dysregulated placental angiogenesis. L-arginine is a precursor of nitric oxide (NO), which plays a central role in promoting endothelial growth, regulating the expression of placental growth factors and angiopoietins (Krause et al., 2011). In Malawi, MiP before 28 GW was associated with lower L-arginine levels and increased levels of NO inhibitors; these in turn, were associated with small for gestational age babies (McDonald et al., 2018). In a mouse model of MiP, supplementation of L-arginine reduced C5 protein and Ang-2 expression, while Ang-1 level was upregulated (McDonald et al., 2018). Furthermore, L-arginine supplementations led to an increase in total number of placental vessel segments and small-diameter vessels (<50 μm) compared to infected controls without supplementation, and this correlated with improved birth weight (McDonald et al., 2018). Of note, these small-diameter vessels are important in vascular remodeling during pregnancy (Geva et al., 2002). Overall, these findings suggest a potential intervention in improving vascularization in the placenta, but whether L-arginine supplementation is useful in pregnant women remains to be investigated.

## Disruption to Placental Development and Utero-Placental Hemodynamics

### The Role of Cytokines

The production of pro-inflammatory cytokines during pregnancy is a double-edged sword. While timely production of these inflammatory mediators assists placental remodeling and growth, dysregulated production may cause irreversible damage to its structure and function. For example, although IFN-γ plays an important role in the remodeling of uterine spiral artery, excessive levels of the cytokine may be detrimental for fetal growth (Ashkar et al., 2000; Suguitan et al., 2003a). In a mouse model of PM, increased IFN-γ levels, and IFN-γ receptor 1 signaling were linked to reduced numbers of vascular branches in the labyrinth of the placentas compared to IFN-γ receptor 1 knocked-out (KO) mice (Niikura et al., 2017). In normal pregnancy, TNF-α expression in the placenta was detectable across all trimesters but was highest in the second trimester (Basu et al., 2016). In MiP, the levels of TNF-α were further elevated and often associated with poor birth outcomes (Rogerson et al., 2003a). Previous *in vitro* studies reported that TNF-α can exert various effects on the placenta such as inhibiting extravillous trophoblast invasion through promoting apoptosis and downregulating human chorionic gonadotropin (hCG) expression by cytotrophoblasts to cause suppressed trophoblast growth and increased apoptosis (Leisser et al., 2006; Otun et al., 2011). These mechanisms could ultimately result in the inhibition of extravillous trophoblast invasion; however, they have yet to be proven in MiP models. Additionally, in a mouse model of PM, increased expression of TNF-α in the placenta was found to induce the expression of tissue factor, which correlated to hemorrhage, fibrin and thrombi formation in the placenta; interestingly, placental architecture can be preserved in mice treated with anti-TNF antibodies (Poovassery et al., 2009).

In *P. falciparum*-infected placentas, increased inflammasome activation was also associated with increase in necrotic areas, fibrioid necrosis and syncytial aggregates (Reis et al., 2020). In the same study, using murine PM model, it was revealed that inflammasome activation led to downstream increase in IL-1β signaling, which contributed to decreased expression of nutrient transporters including SNAT1, SNAT2, and GLUT1 gene expression in *P. berghei*-infected placentas (Reis et al., 2020). In PM, decreased activities of amino acid and glucose transporters have been reported, but it is unclear whether these are similarly mediated through inflammasome activation (Boeuf et al., 2013; Chandrasiri et al., 2014). Furthermore, in mice treated with an IL-1 receptor antagonist or in IL-1β KO mice, nutrient transporter expression was comparable to uninfected controls, suggesting that targeting the IL-1 pathway could be a possible therapeutic approach in MiP (Reis et al., 2020).

### The Role of Placental Hormones and Chemokines

A complex network of placental hormones ensures a functional placenta. Insulin-like growth factor (IGFs) are produced by placental cells and play pivotal roles in placental survival and promoting fetal development (Han et al., 1996). Lack of IGFs was associated with decreased proliferation and poor survival of placental fibroblasts, which can predispose to FGR (Miller et al., 2005; Forbes et al., 2008). In PM, pregnant women were reported to have reduced levels IGF-1, IGF-2, and IL-8, and increased levels of invasion-inhibitory factors including hCG and IL-10 in peripheral blood samples; these factors can inhibit trophoblast invasion and migration (Jovanović et al., 2010; Umbers et al., 2013). In addition, the levels of fetal insulin-like growth factor-binding protein-1 (IGFBP-1) were elevated in PM; this molecule negatively regulates IGFs expression and is associated with placental insufficiency (Shibuya et al., 2011; Umbers et al., 2011; Nawathe et al., 2016). Hypothetically, the dysregulated levels of these growth factors during early trimesters MiP may reduce trophoblast invasion and migration, subsequently affecting the transformation of maternal spiral arteries leading to FGR (Lyall et al., 2013). Healthy placental development also requires chemokines that will recruit immune cells into the decidua during early stage of pregnancy; different chemokines may be needed at different stages of placental development (Ramhorst et al., 2016). However, PM is known to cause elevated levels of chemokines within the intervillous spaces. CCL2 (MCP-1), CCL3 (MIP- 1α), CCL4, CXCL8, CXCL9, CXCL13, and CXCL16 have been observed in the placentas of women with PM, and CXCL9, CXCL13, and CCL4 were associated with adverse pregnancy outcomes such as low birth weight (Ioannidis et al., 2014; Fried et al., 2017). In addition, increased levels of chemokines produced by maternal cells during infection is likely to result in the accumulation of phagocytic cells within the placenta (Suguitan et al., 2003b; Megnekou et al., 2015a). Together, the recruitment of immune cells to the placenta due to infection rather than for the purpose of promoting placental growth, is likely to overdrive inflammation, thus adversely impact on the development of the organ.

### The Role of Complement Proteins

The placenta’s ability to synthesize both complement proteins and their relevant inhibitors suggest that a tightly regulated complement system is essential for a successful pregnancy (Bulla et al., 2009; Lokki et al., 2014). For example, in murine pregnancy model, C1q is synthesized by migrating extravillous trophoblasts to promote trophoblast migration and adhesion, whereas C1q deficiency was associated with impaired labyrinth development (Agostinis et al., 2010). Dysregulated C5a levels may have pathological consequence, as increased C5a expression has been observed in the trophoblasts of pre-eclamptic placentas and C5a-stimulated trophoblasts demonstrated an anti-angiogenic phenotype *in vitro* (Ma et al., 2018). In PM, increased peripheral and placental levels of C5a have been associated with poor birth outcomes in women from all gravidities (Conroy et al., 2009, 2013). In a mouse model of PM, increased C5a and C5a receptor (C5aR) expressions were observed. Interaction between C5a and C5aR was found to impair vascular remodeling necessary to compensate for the placental insult and C5aR blockade improved vasculature development, further highlighting the impact of complement on placental vascularization (Conroy et al., 2013). Of note, these studies were conducted at delivery; hence future studies investigating infection at earlier trimesters are required to further improve understanding on the impact of complement system activation in placental development.

### The Role of Activated Coagulation Cascade

Several studies have shown that MiP induces a pro-coagulative state in the placenta, characterized by perivillous fibrin deposits, elevated biomarkers of coagulation such as tissue factor and D-Dimer, as well as reduced levels of fibrinolysis biomarkers including protein-C, antithrombin-III, and tissue factor pathway inhibitor (Avery et al., 2012; Mostafa et al., 2015). Tissue factor is primarily expressed by macrophages that are found in abundance in *P. falciparum*-infected placentas, particularly those with chronic PM, and may be the reason for increased fibrin deposition in these placentas (Hohensinner et al., 2021). High levels of plasminogen activator inhibitor-1 (PAI-1) can suppress fibrinolysis, leading to increased fibrin deposition and pathological effects on tissues. Interestingly, pregnant women with submicroscopic infections, presumably due to having lower parasite density, had increased levels of PAI-1 but not fibrin deposition; PAI-1 levels were comparable to that of PM detected by microscopy (Avery et al., 2012). The authors also found low levels of inflammation in the submicroscopic cases, suggesting that fibrin deposition/coagulation is likely to be triggered only in chronic infection. Indeed, increase in fibrin deposits was more common in placentas from primigravids, who are usually unable to effectively clear infection due to lack of immunity (Rogerson et al., 2003b). Given that coagulation may play a role in placental pathology, therapeutics that target the inflammatory-coagulation pathways may be useful in preventing placental injury. *In vivo*, the treatment of *P. chabaudi*-infected pregnant mice with low molecular heparin (an anticoagulant), showed absence of placental hemorrhage, tissue necrosis and large fibrin deposit in placentas (Avery et al., 2012). More studies are required to determine if similar effects can be achieved in humans.

## Dysregulated Autophagy and Heat Shock Proteins in Rescue Mechanisms

Autophagy is an important mechanism in normal pregnancy as it plays an important role in embryogenesis, implantation and placentation (Nakashima et al., 2019). Autophagy is activated in extravillous trophoblasts during the invasion and vascular remodeling process early in pregnancy. In addition, it appears to be a protective mechanism against cellular senescence in trophoblasts (Burton et al., 2017). However, increased autophagic activities in the villous trophoblasts have been associated with FGR and preterm labor (Nakashima et al., 2019). In placental biopsies, PM with intervillositis increased autophagosome (an upstream pathway of autophagy) formation but not autophagy, and the density of autophagosome formation correlated negatively with amino acid uptake, suggesting that impaired autophagy may reduce transplacental amino acid transport (Dimasuay et al., 2017b). In another study, mRNA expression of autophagic genes were reduced in women with PM and was associated with low birth weight delivery (Lima et al., 2019). The triggers of autophagic responses in MiP remains unclear, although hypoxia or inflammation are likely candidates (Oh and Roh, 2017). The effect of MiP on autophagy in earlier stages of pregnancy is also unknown, as current studies employed the use of term placentas.

Heat shock proteins (HSPs) are produced by cells to promote recovery from an injury or stress. HSPs 27, 60, 70, and 90 are also found to be in abundance in the endometrial and uterine cells especially in the first trimester, suggesting that they may be involved in placental development (Jee et al., 2021). Their roles in pregnancy may include maintaining the integrity of decidual cells, promote syncytialization and provide protection from preterm deliveries (Jee et al., 2021). On the other hand, elevated levels of certain HSPs have been associated with adverse pregnancy outcomes including FGR and preterm deliveries, suggesting that abnormally high levels of HSPs may indicate excessive tissue damage (Tan et al., 2007). In *P. berghei* PM model, levels of HSP 90, 70, 60, and 25 were increased in the placenta during infection, and levels of HSPs 70, 60, 25 but not HSP 90 decreased gradually with increasing disease severity (Sharma et al., 2014). The decrease in HSPs 70, 60, and 25 was proposed to be responsible for placental necrosis, while increased HSP 90 appears to be a compensatory mechanism to repair damaged placental cells (Sharma et al., 2012, 2014). The mechanisms leading to altered HSP levels and how the repair mechanisms of these proteins are affected in MiP require further attention.

### *In vitro* and *in vivo* Models to Delineate Pathways in the Placenta During Malaria in Pregnancy

Models that can recapitulate features of MiP are required for studying mechanisms underlying placental pathologies, as well as for testing potential therapies. Monolayer cell lines including primary human trophoblast, Swan 71 and BeWo cells have been used in MiP research, and some findings from these 2D models were successfully translated to *in vivo* findings (Boeuf et al., 2013; Umbers et al., 2013; Dimasuay et al., 2017a). However, there are still major limitations associated with these models; continuous cell lines have chromosomal abnormalities and single-cell model lacks the immunological stimuli that are usually present in the placentas during PM. Pehrson et al. (2016) reported that a novel *ex vivo* placental perfusion model can be used to investigate receptor-ligand interactions and they demonstrated the accumulation of parasites in the placenta. This may be a useful model to study real-time pathological changes in the placental tissues at later stage in pregnancy, albeit with some limitations such as requiring highly skilled personnel and specialized equipment. *In vivo* rodent models have been used to study PM, but their transferability to humans remains an issue. Mouse models were able to replicate certain consequences of PM including dysregulated immunological factors, imbalanced angiogenesis and vasculogenesis factors, and poor outcomes in offsprings (Megnekou et al., 2013; Doritchamou et al., 2017; McDonald et al., 2018). Additionally, structural similarities and presence of analogous placental cell types between rodents and humans make them an attractive model for MiP (Georgiades et al., 2002). However, there are differences in their placental structures, and certain features of placental inflammation such as monocytes/macrophages accumulation were not seen (Poovassery and Moore, 2006; Sharma et al., 2016). In addition, the lack of hemozoin accumulation in mouse placentas suggests that the model may be suitable for studying acute rather than past and/or chronic infection (Boareto et al., 2019). There are other limitations associated with rodent models including the absence of pre-existing immunity in mice which is not reflective of the conditions of pregnant women living in malarious regions, as well as difference in the degree of parasite sequestration and mode of infection between rodents and humans (Table 1). On the other hand, while non-human primate models are more similar to humans in terms of physiology, high cost and sustainability issues arises. The suitability of animal models in MiP has been extensively discussed in many recent reviews, thus will not be further discussed here (Doritchamou et al., 2017; Barateiro et al., 2019).

MiP in the first trimester can cause suboptimal development and irreversible damage to the placenta, hence it is crucial to study the effects of MiP during early pregnancy (Chaikitgosiyakul et al., 2014; Moeller et al., 2019; Obiri et al., 2020). However, this proves to be a huge challenge, as existing models fail to replicate infection during early trimester. In recent years, newer models such as placental organoids have been used to study the placenta. Organoids are miniaturized *in vitro* tissue construct that are isolated from stem cells and the *in vitro* culture is usually representative of the organ *in vivo*. Placental organoids can be isolated from first-trimester trophoblasts and with appropriate growth factors and conditions, they can differentiate into SCT and extravillous trophoblasts that closely resemble first trimester placentas (Haider et al., 2018; Okae et al., 2018; Turco et al., 2018). This would provide vast experimental opportunities on two major trophoblast layers that are involved in early placental development. For example, a recent study was able to establish innate immune signaling pathways during Zika infection using maternal-derived organoids and key roles of antiviral immunity at the maternal-fetal interface can be elucidated (Yang et al., 2021). The use of trophoblast organoids to study early impact of MiP would allow real-time precise identification of placental responses during infection. Experimental design that includes a co-culture system of the organoids with immune cells can provide a better representation of the placental response, and specific cell populations that are responsible for placental damage or impaired placental development can be identified. This model can also be considered for the study of cytotoxicity and efficacy of potential therapeutics for early trimester MiP.

## Conclusion

MiP remains a huge threat to the well-being of pregnant women and their developing fetus in malaria-endemic regions. Pregnant women are at the highest risk of MiP in the first trimester, which often results in poor placental development and irreversible placental structure damages that contribute to poor birth outcomes. Dysregulated levels of various soluble mediators have been associated with placental pathologies, including cytokines, chemokines, complement proteins, and growth factors. However, our current understanding of the pathogenetic mechanisms in the placenta relies either on a single time point during pregnancy, usually at delivery, or on animal models, which have their own limitations. Hence, there is a need to develop better models of the placenta to obtain a more comprehensive understanding of the pathogenesis of MiP during early pregnancy and follow through the disease progression. These efforts will ultimately enable the design of targeted strategies to aid placenta recovery and minimize the impact of MiP.

## Author Contributions

All authors listed have made a substantial, direct and intellectual contribution to the work, and approved it for publication.

## Conflict of Interest

The authors declare that the research was conducted in the absence of any commercial or financial relationships that could be construed as a potential conflict of interest.

## Publisher’s Note

All claims expressed in this article are solely those of the authors and do not necessarily represent those of their affiliated organizations, or those of the publisher, the editors and the reviewers. Any product that may be evaluated in this article, or claim that may be made by its manufacturer, is not guaranteed or endorsed by the publisher.
